# Urine NGAL adds to serum creatinine in predicting cefepime clearance in critically ill children at high risk of acute kidney injury

**DOI:** 10.1016/j.ijantimicag.2026.107741

**Published:** 2026-02-12

**Authors:** Horace Rhodes Hambrick, Ronaldo Morales, Calise Curry, Michaela Collins, Luana Johnson, Tomoyuki Mizuno, Kelli A. Krallman, Stuart L. Goldstein, Sonya Tang Girdwood

**Affiliations:** a Division of Nephrology, Ann & Robert H. Lurie Children’s Hospital of Chicago, Chicago, IL, USA; b Department of Pediatrics, Northwestern University Feinberg School of Medicine, Chicago, IL, USA; c Division of Translational and Clinical Pharmacology, Cincinnati Children’s Hospital Medical Center, Cincinnati, OH, USA; d Division of Hospital Medicine, Cincinnati Children’s Hospital Medical Center, Cincinnati, OH, USA; e Center for Acute Care Nephrology, Cincinnati Children’s Hospital Medical Center, Cincinnati, OH, USA; f Department of Pediatrics, University of Cincinnati College of Medicine, Cincinnati, OH, USA; g Division of Nephrology and Hypertension, Cincinnati Children’s Hospital Medical Center, Cincinnati, OH, USA

**Keywords:** Beta-lactam pharmacokinetics, Acute kidney injury, Urine biomarkers, Model-informed precision dosing, Monte Carlo simulations

## Abstract

**Introduction:**

Cefepime clearance (CL) depends on renal function, but serum creatinine (SCr) lags behind real-time changes in kidney function. Urinary neutrophil gelatinase–associated lipocalin (uNGAL) is a biomarker of real-time tubular injury, though its role in predicting cefepime pharmacokinetics (PK) is unclear.

**Methods:**

We conducted a prospective study in paediatric intensive care unit (PICU) patients at high risk for acute kidney injury who received cefepime within 48 h of admission and had uNGAL measured. A validated population PK model incorporating SCr-estimated GFR (eGFR) was adapted to test the added predictiveness of uNGAL. Inter-occasion variability (IOV) assessed whether CL and central volume (V1) differed after 48 h of PICU admission. Monte Carlo simulations ( *n* = 10 000) evaluated the probability of pharmacodynamic (PD) target attainment (PTA) for various cefepime regimens and PK/PD targets across weight groups (5–40 vs. 40–100 kg), kidney function strata (eGFR 30–60, 60–90, 90–150 mL/min/1.73m^2^), and uNGAL thresholds (≥500 vs. < 500 ng/mL).

**Results:**

Fifty patients (median age 11.5 y) were enrolled. PICU admission uNGAL ≥500 ng/mL was associated with ~35% lower CL, independent of eGFR, with IOV at 4 h post-PICU admission significant for CL and V1. Patients with low uNGAL had higher CL at a given eGFR with consequently lower PTA for a given dosing regimen, necessitating extended or continuous infusions to reach PK/PD targets.

**Conclusions:**

Integrating uNGAL improves the prediction of cefepime CL beyond SCr alone. Patients with low uNGAL despite elevated SCr risked subtherapeutic exposure. Urine biomarker-guided dosing may help optimise PTA and warrants further study.

## 1. Introduction

Cefepime, a fourth-generation cephalosporin, is widely used in critically ill paediatric patients with or at-risk for bacterial sepsis due to its broad-spectrum antimicrobial activity. Its pharmacokinetics are heavily dependent on renal function, with renal clearance (CL) accounting for ~85% of its elimination [1]. Pharmacokinetic/pharmacodynamic (PK/PD) targets for beta-lactam antibiotics like cefepime often emphasise maximizing the percent of time free concentrations exceed bacterial minimum inhibitory concentrations (MIC) throughout the dosing interval (% *f*T > MIC) [2,3]. However, debates persist regarding optimal PD targets, ranging from 50% *f*T >MIC to a more aggressive goal of 100% *f*T > 4x MIC [4]. Nonetheless, providing adequate cefepime exposure is critical to maximise antimicrobial effect, particularly given the importance of adequate antibiotic exposure within the first 48 h of sepsis to improving outcomes [5].

On the other hand, excessive cefepime concentrations have been associated with neurotoxicity, with symptoms ranging from agitation and tremors to seizures and coma, predominantly documented in adult populations [6-8]. Paediatric data on cefepime-induced neurotoxicity are scarce, limited mostly to case reports, and definitive neurotoxicity thresholds remain undefined [9-12]. Paediatric cefepime neurotoxicity has been described exclusively in patients with kidney dysfunction, presumably because, since cefepime is renally eliminated, children with kidney dysfunction are at risk of excessive exposure to cefepime or a neurotoxic metabolite. Therefore, identification of factors predicting cefepime CL in patients at high risk of kidney dysfunction has the potential to ensure adequate provision of drug exposure while avoiding potential toxicity.

Acute kidney injury (AKI) occurs in one-third of critically ill children and alters cefepime clearance significantly [13]. Serum creatinine (SCr), the standard marker used to evaluate kidney function, has limitations because it lags behind real-time changes in glomerular filtration rate (GFR) [14]. In addition, given the curvilinear relationship between SCr and GFR, relatively small changes in creatinine at low absolute values of creatinine reflect large changes in GFR [15]. Moreover, given the kidney functional reserve present in most patients, with the ability of renal autoregulation to maintain GFR in the setting of stressors, there may be a substantial loss of functional kidney tissue before SCr changes [16]. Thus, prior investigations have identified tubular injury biomarkers that can identify so-called subclinical AKI (AKI stage 1S), or kidney damage that has occurred before a change in eGFR biomarkers like SCr [17– 19]. One such biomarker is urine neutrophil gelatinase-associated lipocalin, or uNGAL, a protein produced by kidney cells and leukocytes in the context of kidney tubular injury [20,21]. Elevations in uNGAL have been shown to predict subsequent AKI in multiple populations, with uNGAL elevations occurring before changes in SCr [20-24].

While changes in uNGAL may predict consequent AKI, it is unknown to what extent these changes in uNGAL are consistently predictive of changes in the clearance of drugs that have significant renal elimination, such as cefepime. Previous work has demonstrated that uNGAL is more strongly associated with tobramycin clearance than SCr [25] and that low uNGAL in critically ill children receiving cefepime is associated with failure to attain the PD target of 100 percent of time free concentrations exceed minimum inhibitory concentration (% *f*T > MIC) using an MIC of 8 mg/L for *Pseudomonas aeruginosa*, with a median uNGAL of 12 ng/mL among patients with a cefepime C_min_ of <8 mg/L and a median uNGAL of 350 ng/mL among those with a C_min_ of >8 mg/L [26]. This suggests that lower uNGAL, which is indicative of the absence of kidney injury, may be associated with higher cefepime clearance and, therefore, potential failure to achieve PD targets. On the other hand, a 2018 study of urinary biomarkers after cardiac surgery-associated AKI showed that elevated urine kidney injury molecule-1 (KIM-1) and the product of urine tissue inhibitor of metalloproteinase-2 and insulin-like growth factor-binding protein 7 (TIMP-2*IGFBP7) were associated with increases in serum milrinone concentrations before changes in SCr, but elevations in uNGAL were not [27]. Therefore, it is not known whether uNGAL reliably predicts changes in drug PK beyond or before changes in SCr.

This study aimed to evaluate whether incorporating uNGAL with SCr assessments improves predictions of elevated cefepime exposure in critically ill paediatric patients at high risk of AKI during the early phase (i.e. first 48 h of PICU admission) and later phase of PICU admission.

## 2. Methods

### 2.1. Study design and patient population

We conducted a prospective observational study enrolling paediatric patients admitted to the PICU with an elevated risk of acute kidney injury defined by an elevation in the renal angina index (RAI) of 8 or greater, prescribed cefepime with a start date up to 48 h after PICU admission, and who had a uNGAL obtained as part of standard of care. Cincinnati Children’s Hospital Medical Center (CCHMC) has systematically incorporated routine testing of uNGAL in patients at high risk of severe (Kidney Disease Improving Global Outcomes [KDIGO]-defined Stage 2–3 [28]) AKI (sAKI), using a renal angina index (RAI)-based protocol. As described previously, the RAI is calculated at 12 h after PICU admission and uses a combination of risk factors for AKI (solid organ or bone marrow transplant, intubation, and/or use of vasopressors) with evidence of existing injury (elevations in SCr and/or fluid overload) to calculate a patient’s risk of sAKI [29]. Previous work has shown that an RAI of <8 has a negative predictive value of 0.97 for ruling out sAKI at day 3 of ICU admission, while an RAI of ≥8 and uNGAL of ≥150 ng/mL increases the positive predictive value of RAI for sAKI from ~0.3 (for RAI of 8 alone) to 0.64 [24]. Thus, patients with an RAI of 8 or greater have a reflex order released for a uNGAL to be obtained.

Patients who were already receiving dialysis or extracorporeal therapies (e.g. extracorporeal membrane oxygenation, continuous kidney replacement therapy, haemodialysis, peritoneal dialysis, or therapeutic plasma exchange) at the time of screening were excluded; data from patients who started these therapies after study enrolment were included only prior to starting these therapies. The study was approved by the CCHMC institutional review board (IRB) with a waiver of consent (IRB #2018-0724).

Study Day 1 was defined as the first day of cefepime administration within 48 h before PICU admission, up to 48 h after PICU post-admission. As this was an observational study, patients received the cefepime doses and frequencies prescribed by their clinical team. At our institution, cefepime is typically prescribed at 50 mg/kg/dose (maximum 2000 mg/dose) as a 30-min infusion every 8 h for those with normal kidney function (eGFR > 60 mL/min/1.73 m^2^), every 12 h for those with moderately decreased kidney function (eGFR 30–60 mL/min/1.73 m^2^), and every 24 h for those with severely decreased kidney function (eGFR < 30 mL/min/1.73 m^2^), with eGFR determined by the treating team.

### 2.2. Blood sampling and cefepime concentration assessment

As previously described, residual blood samples from routine care were scavenged for cefepime concentration measurement [30]. Our research group has previously employed this approach and demonstrated stability of cefepime in these samples for up to 72 h at 4 °C [30]. Samples in these studies were stored under the same conditions for no longer than 72 h before centrifugation (2060 × *g*, 4 °C, 10 min). The supernatant was stored at −80 °C for up to 180 d until total cefepime concentrations were measured via high-performance liquid chromatography (HPLC). Our research group has previously described the HPLC assay to quantify cefepime concentrations, noting that the assay range exhibited linearity from 0.5 to 200 mg/L [30]. This assay’s precision and accuracy have coefficients of variation maintained below 15%.

### 2.3. Clinical data collection

We reviewed electronic medical records to collect clinical and demographic data for up to nine study days. Data were stored in a secure REDCap database [31]. We calculated the estimated glomerular filtration rate (eGFR) using the bedside Schwartz equation for patients aged <18 y and the race-neutral SCr-based chronic kidney disease epidemiology collaboration (CKD-EPI) equation for patients aged ≥18 y [32,33]. We quantified fluid status by tabulating daily net fluid intake and output data to calculate daily net fluid balance (daily intake − daily output from midnight to midnight). We then defined the cumulative percentage of fluid balance (CumFB) as the sum of all the previous days’ and present day’s percentage of fluid balance (sum of each day’s net fluid balance/body weight on study day 1 × 100). We handled missing data by using the ‘last observation carried backwards’ method, in which the newest data were extended to fill the missing data. uNGAL and SCr values below the limit of quantification were assigned the value of the lower limit of quantification (50 ng/mL for uNGAL and 0.1 mg/dL for SCr). AKI was defined by standard KDIGO creatinine-based criteria [28] by assuming the patient’s baseline SCr as the lowest value available within 90 d prior to admission or imputing a normal SCr for age and height by the bedside Schwartz formula, assuming a normal eGFR of 120 mL/min/1.73 m^2^ if no baseline SCr is available, as described previously [34].

### 2.4. Population pharmacokinetic modelling

To investigate whether uNGAL is a significant predictor of cefepime clearance, we tested uNGAL as a new covariate in a cefepime population pharmacokinetic (popPK) model previously developed and validated in our PICU patients, which originally included SCr-eGFR as a major predictor of cefepime clearance [35]. Briefly, it is a two-compartment model that incorporates allometrically scaled body weight to account for size differences, with eGFR as a covariate on clearance and cumulative fluid balance as a covariate on central volume of distribution (V1). We selected this model as the base since it was built using data from patients in the same PICU and had already undergone external validation using a subset of patients from the present study [35]. All PK analyses were performed using Monolix Suite software (2024R1 Version, Lixoft, Antony, France).

As part of our exploratory data analysis, we generated individual PK parameter predictions using the original popPK model, primarily to visually investigate the relationship between individual cefepime CL and uNGAL. Multiple approaches were explored to incorporate uNGAL into the model. Given the right-skewed distribution of uNGAL values, we evaluated both log-transformed uNGAL and inverse uNGAL (1/uNGAL) as continuous covariates. We also tested PICU admission uNGAL as a categorical covariate using cut-offs of 150, 500, and 1000 ng/mL. In all model iterations, PK parameters other than tested covariate effects were fixed to the values estimated in the original cefepime popPK model, as previously described [35]. This was done in an effort to isolate the incremental effect of uNGAL on clearance and to reduce the risk of overfitting. The only exception was the beta coefficient corresponding to eGFR’s effect on clearance, which was re-estimated in each model to account for potential collinearity with uNGAL. Parameter estimation was performed using the SAEM (Stochastic Approximation Expectation-Maximisation) algorithm in Monolix to provide maximum likelihood estimates.

We also hypothesised that the first 48 h of PICU admission would be pharmacokinetically distinct. This is because organ function is dynamic, both from the impact of critical illness and the large volumes of fluid often used in the resuscitative period in early sepsis, and beta-lactam PK has been shown to differ before vs. after 48 h of PICU admission [36]. In addition, the first 48 h of ICU admission in patients with sepsis represent a critical period where adequate PD target attainment is critical due to a high risk of death [5]. Thus, we assessed the impact of time relative to PICU admission by defining the data within the first 48 h of PICU admission as one occasion and the data thereafter as a second occasion, estimating potential inter-occasion variability (IOV) on both CL and V1. As in the base model, sparse opportunistic sampling precluded reasonable estimation of random effects on intercompartmental clearance (Q) and the peripheral volume of distribution (V2). uNGAL was *a priori* retained as a covariate in the final model if its inclusion resulted in a decrease in the objective function value (OFV) of at least 6.63 ( *P* < 0.01) and if the covariate effect could be estimated with a relative standard error of below 40%. Because uNGAL was evaluated in multiple forms (e.g., log-transformed, inverse, and categorical), we compared all qualifying models and retained the version that produced the greatest reduction in OFV while satisfying these inclusion criteria. The final model was then validated through inspections of individual fit plots and goodness-of-fit plots, including observed vs. individual and population-predicted concentrations, and scatter plots of the residuals. We then performed a prediction-corrected visual predictive check (pcVPC) with 500 simulations per Monolix default and a 1000-fold non-parametric bootstrap analysis from random sampling from the original dataset, and the median and 95% confidence intervals of the parameter estimates were compared with the final model estimates.

### 2.5. Monte Carlo simulations

To estimate the impact of elevations in uNGAL in predicting PD target attainment, we performed Monte Carlo simulations by implementing the final model in Simulx software (2024R1 version, Lixoft, Antony, France).

We created two simulation datasets comprising 10 000 patients each, one sampled randomly from a uniform distribution of weights from 5 to 40 kg (weight-based dosing of 50 mg/kg/dose) and another sampled randomly from a uniform distribution of weights from 40 to 100 kg (fixed dosing of 2000 mg per dose). We then used these weight distributions to create three sub-populations to simulate moderately decreased kidney function (eGFRs randomly sampled from a uniform distribution from 30 to 60 mL/min/1.73 m^2^), low-normal kidney function (eGFR 60–90), and high-normal kidney function (eGFR 90–150). We then assigned each of these six sub-populations a uNGAL ≥500 ng/mL (elevated uNGAL) for one set of simulations and a uNGAL <500 ng/mL for the subsequent set. The cumulative percentage of fluid balance was set to 0% for all patients.

The probability of target attainment (PTA) was assessed using simulated concentration-time profiles at 48 h for six different cefepime dose regimens: 50 mg/kg (maximum 2000 mg/dose) every 8 or 12 h, administered with a 30-min or 3-h infusion duration, then 100 mg/kg/d (maximum 4000 mg) and 150 mg/kg/d (maximum 6000 mg) as continuous infusions over 24 h. We sought to identify the dosing regimens that would ensure that at least 90% of simulated patients had free concentrations that met two distinct PD targets: 100% *f*T > MIC and 100% *f*T > 4x MIC, using the 2025 Clinical & Laboratory Standards Institute cefepime breakpoints of MIC 2 mg/L for *Enterobacteriaceae* and MIC 8 mg/L for *Pseudomonas aeruginosa* [37]. We assumed 20% protein binding to estimate free cefepime concentrations from the total concentrations measured [38].

## 3. Results

### 3.1. Patient characteristics

Fifty patients (median age 11.5 y [IQR: 5–17; range: 3 months–31 y], 50% female sex; see Table 1 for full descriptive statistics) with a total of 296 cefepime concentrations were included, 175 (59%) of which were collected within 48 h of PICU admission. Individual patients had 2–11 (median 6) cefepime concentrations collected. Overall illness severity was high, with 33 patients (66%) requiring pressors and 37 (74%) requiring mechanical ventilation (new or with settings above baseline support) during their PICU stay; 48 (96%) had at least one organ failure (see definitions in Table 1) during their PICU admission with a median of 2 (IQR 2– 3) organ failures per patient. Median SCr-eGFR on PICU admission was 62 mL/min/1.73 m^2^. All patients had at least one SCr and uNGAL measured during their PICU admission. Thirty-three (66%) patients had SCr-defined KDIGO Stage 2 or more AKI on PICU admission. Twenty-five (50%) of all patients, regardless of AKI status (including none), had an elevated uNGAL using a threshold of 150 ng/mL, and 15 (30%) had an elevated uNGAL using a threshold of 500 ng/mL.

### 3.2. Microbiology data

The majority of patients received cefepime as empiric therapy; only 10 (20%) patients had a positive blood, urine, or respiratory culture Table S1).

### 3.3. Pharmacokinetic analysis

**Fig. 1.**
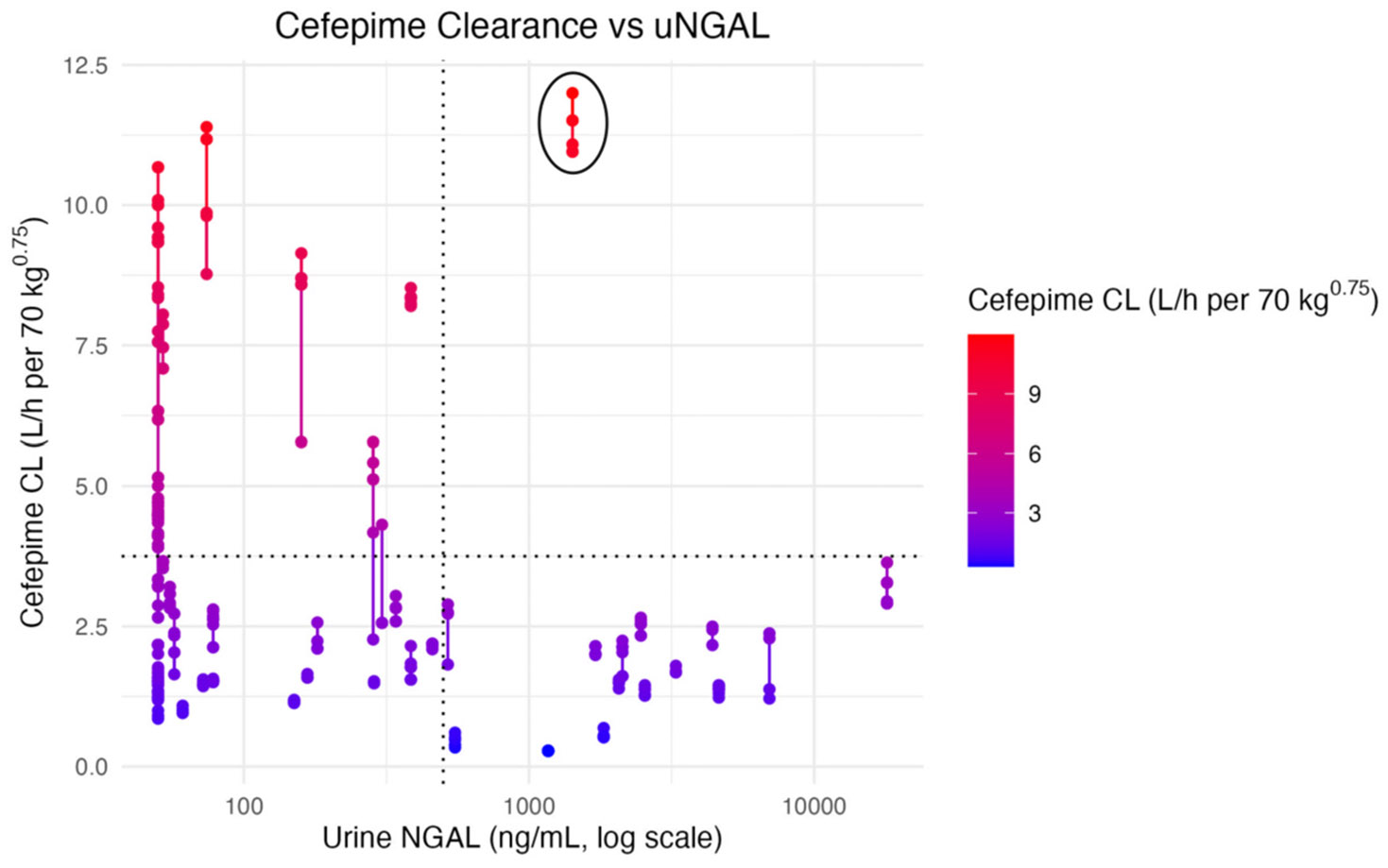
Individual cefepime clearance vs. uNGAL at PICU admission in patients with high risk of AKI. CL, clearance; h, hour; L, litres; uNGAL, urine neutrophil gelatinase-associated lipocalin. Dotted lines represent the uNGAL threshold of 500 ng/mL (vertical) and clearance threshold of 3.75 L/h/70 kg^0.75^ (horizontal). Individual dots represent clearance estimates based on a particular concentration, with the lines between dots connecting the change in clearance estimates over time for an individual patient. The patient who was excluded from further analysis was the single patient circled in the top right quadrant with clearance estimates above 3.75 L/h/70 kg^0.75^ at any point during PICU admission despite a uNGAL >500 ng/mL.

Visual inspection of uNGAL vs. model-predicted individual cefepime clearance, using the model before uNGAL inclusion (Fig. 1), showed that all but one patient’s clearance estimates for patients with a uNGAL >500 ng/mL were <3.75 L/h/70 kg^0.75^. This patient had paraplegia and had a SCr of 0.35 mg/dL despite a height of 185 cm, resulting in a supra-normal eGFR of ~200 mL/min/1.73 m^2^. Given this, he presumably had abnormally low muscle mass, leading to a low SCr and, therefore, over-estimation of SCr-eGFR and, consequently, of drug CL; this patient’s data were excluded from the final analysis.

Modelling uNGAL as a binary variable using a threshold of 500 ng/mL yielded the greatest reduction in the OFV, met statistical criteria for inclusion ( ΔOFV > 6.63, P < 0.01), and had an effect estimate with acceptable precision (relative standard error <40%), leading to its inclusion in the final model. In addition, incorporating IOV, defined as the first 48 h of PICU admission and after, on both CL and V1 improved model fit ( ΔOFV > 6.63, *P* < 0.01), supporting its inclusion. Consideration of early (before 48 h) vs. late (after 48 h) as a potential predictor of clearance was not significant, suggesting that there was not a consistent effect in one direction, i.e., for some patients, CL increased after 48 h, while for others, it decreased; regardless, we do know there was variability in CL within individual patients when considering the first 48 h of PICU admission compared with the time thereafter. The final PopPK model is presented in Table 2.

Model performance was supported by diagnostic plots presented in Figs. S1-S3. Goodness-of-fit plots showed no significant evidence of model misspecification, with observed values symmetrically clustered around the line of identity and residuals centred around zero. The pcVPC demonstrated that the 2.5th, 50th, and 97.5th percentiles of observed concentrations were well contained within the 95% prediction intervals. The bootstrap analysis confirmed the stability of covariate effect estimates, with median estimates and 95% confidence intervals consistent with the final model (Table 2).

### 3.4. Monte Carlo simulations

Results from Monte Carlo simulations are available in Tables 3 and 4 , with a summary of corresponding dosing recommendations in Table S2.

When considering patients 5–40 kg (weight-based dosing, Table 3), 50 mg/kg every 12 h as a 3-h infusion (extended infusion, EI) was adequate for 90% PTA for the target of 100% *f*T > 1xMIC of 8 mg/L (*Pseudomonas* breakpoint) for patients with an eGFR 30–60 mL/min/1.73 m^2^ and uNGAL ≥500 ng/mL, but 50 mg/kg every 8 h as an EI was necessary to achieve this target for patients with the same eGFRs but a uNGAL <500 ng/mL. For 100% *f*T >4x MIC of 8 mg/L, 100 mg/kg/d as a continuous infusion (CI) was adequate when uNGAL was ≥500 ng/mL, but 150 mg/kg/d as a CI was needed when uNGAL <500 ng/mL. Similarly, for patients with an eGFR 60–90 mL/min/1.73 m^2^, 50 mg/kg every 8 h as a 30-min infusion (standard infusion, SI) was adequate for 90% PTA for 100% *f*T > 1 x MIC 8 mg/L when uNGAL ≥500 ng/mL; 100 mg/kg/d as a CI was needed when uNGAL <500 ng/mL and no regimen tested achieved 100% *f*T >4x MIC of 8 mg/L. For those with an eGFR of 90–150 mL/min/1.73 m^2^, 50 mg/kg every 8 h as an EI was adequate for 90% PTA for 100% *f*T > 1x MIC 8 mg/L when uNGAL ≥500 ng/mL and 150 mg/kg/d as a CI would achieve 100% *f*T > 4x MIC 8 mg/L, but when uNGAL <500 ng/mL, 100 mg/kg/d as a CI was needed to achieve 100% *f*T > 1x MIC 8 mg/L and no regimen tested achieved 100% *f*T > 4x MIC of 8 mg/L.

Target attainment was generally higher in the fixed-dose (40–100 kg) cohort (Table 4), though a similar pattern emerged in which patients with an elevated uNGAL had higher PTA for a given eGFR. In this group, 6000 mg/d as a CI was adequate to achieve 90% PTA for 100% *f*T >4x MIC of 8 mg/L with eGFR 30–90 mL/min/1.73 m^2^ and uNGAL ≥500 ng/mL, but none of the tested regimens achieved 90% PTA for the PD target of 100% *f*T >4xMIC of 8 mg/L when eGFR >90 mL/min/1.73 m^2^ or uNGAL <500 ng/mL regardless of eGFR.

## 4. Discussion

Herein, we demonstrate that a uNGAL concentration of ≥500 ng/mL obtained on PICU admission is associated with a significant decrease in cefepime clearance even after accounting for estimated kidney function using SCr-defined eGFR. Specifically, this elevated uNGAL on PICU admission was associated with an approximately 35% decrease in cefepime clearance throughout PICU admission. This study underscores the potential clinical utility of tubular injury biomarkers such as uNGAL in enhancing the prediction of cefepime pharmacokinetics in critically ill children with AKI. Given cefepime’s renal clearance and the limitations of SCr-based AKI assessments, integrating tubular biomarkers may allow for personalised drug dosing strategies, particularly at institutions where clinical monitoring of cefepime concentrations is not available. uNGAL is currently used in the CCHMC PICU to predict patients who are at high risk of severe persistent AKI. That is, based on results from the TAKING-FOCUS 2 study [29], patients who have an elevated RAI and uNGAL ≥150 ng/mL have a ~68% chance of developing severe persistent acute kidney injury, while those who had an elevated RAI but NGAL <150 reduced the risk of ever developing severe AKI to 29% [24,29]. Our study adds to TAKING FOCUS 2 by demonstrating that drug PK follows a similar pattern: those with an elevated SCr and elevated uNGAL had decreased cefepime clearance compared with those who had elevated SCr but low uN-GAL.

Clinicians are typically familiar with reducing the dose or frequency of medication in patients with kidney dysfunction based solely on SCr-eGFR. However, we show that within these eGFR-based groups, there is variability in cefepime clearance that can be partially accounted for by uNGAL. Using the same dosing regimen for all patients in an eGFR-based group could risk overexposure in some or underexposure for others. For example, for a child with eGFR between 30 and 60 mL/min/1.73m^2^, 50 mg/kg every 12 h over 3 h may meet the target of 100% *f*T >8 mg/mL when uN-GAL ≥500 ng/mL, but may lead to underexposure in a patient with uNGAL <500 ng/mL. In other words, patients with elevated SCr but uNGAL <500 ng/mL likely have a temporary, reversible, functional AKI rather than a more sustained structural AKI and may benefit from more frequent cefepime dosing and/or extended infusions to ensure adequate PD target attainment. Conversely, some patients with ‘normal’ SCr-eGFR might need dose or frequency reduction when uNGAL is high, and failing to do so may lead to unnecessarily high concentrations. That is, the goal of incorporating uNGAL was to identify patients within SCr-eGFR strata who are at risk of relative under- or over-exposure, thereby facilitating provision of the minimum effective dose of cefepime. Indeed, aiming for the minimum necessary dose for PD target attainment is critical since rigorous prospective evaluations of cefepime-related neurotoxicity in paediatric populations are lacking, and exact neurotoxicity thresholds in children are unknown [9,11,12].

This study is notably concordant with the findings of a recent study of uNGAL and cefepime PK in critically ill children, which similarly found that low uNGAL and low urine KIM-1, though not urine osteopontin or urine cystatin C, were associated with failure to achieve PD targets within the first 48 h of cefepime initiation.[26] Our study adds to this by corroborating this association with uNGAL in a larger cohort and by quantifying this relationship through population PK modelling. In addition, by quantifying this relationship using population PK modelling, we provide practical, model-informed strategies for dose adjustment based on uNGAL levels. While prior studies have suggested that uNGAL is a sensitive early marker of kidney injury, dosing decisions in clinical practice still rely primarily on SCR-eGFR because there is no actionable guidance for using uNGAL in dosing, which our study addresses.

Strengths of this investigation include its prospective design and proof of the benefit of utilising a reliable biomarker already used in clinical practice in predicting drug PK. That is, a recent study of uNGAL in real clinical practice showed a test-retest coefficient of variation of <5% and stability in room temperatures for up to 48 h [39]. Moreover, the assay takes less than 10 min to run and has been successfully integrated into an AKI risk prediction algorithm at our institution, underlying its ease of use. In addition, despite using only opportunistic samples, we were able to characterise pharmacokinetics both before and after 48 h of PICU admission. Therefore, the results of this study provide readily actionable guidance for adjusting dosing based on a simple urine biomarker test.

This study is not without limitations. As an observational study from a single centre, we could not test the real-life performance of dosing regimens other than those prescribed by the clinical team, and we did not have a systematic or longitudinal collection of uNGAL. Additionally, since uNGAL concentrations may vary based on the degree of overall urinary concentration (and thus urine volume), normalising uNGAL to urine creatinine, which also varies based on urine concentration, may have strengthened the associations seen. However, urine creatinine values were not available for most patients, and studies of uNGAL as a potential predictor of AKI have typically incorporated uNGAL in absolute terms, not normalised to urine creatinine. We also excluded anuric patients (and therefore unable to produce a uNGAL) or receiving dialysis, phere-sis, or other extracorporeal therapies, all of whom have variable PK and would benefit from additional studies. In addition, since we mostly included patients with kidney injury or high risk of kidney injury, many of our patients had a low eGFR, so our predictions for patients with high-normal kidney function are based partly on extrapolation. We also have not externally validated the model. Moreover, since the cefepime neurotoxicity threshold for children (and what PK parameter with which to quantify this) is unknown, we could not comment on the risk of neurotoxicity with the proposed dosing regimens. Finally, while we chose to assess target attainment 48 h after PICU admission since prompt antibiotic administration during the first 48 h of sepsis is critical to improving outcomes, not all patients were septic at the time of PICU admission or had an identified infection, so the physiologic changes seen in early sepsis might not have been present in all patients during this period. Nevertheless, the clear signal for IOV at this 48-h cut-off underscores the validity of this timepoint notwithstanding this limitation.

## 5. Conclusions

In summary, elevated uNGAL on PICU admission predicts a decrease in cefepime CL even after accounting for SCr-eGFR, and patients with a low uNGAL may be at risk of inadequate pharmaco-dynamic target attainment. These findings provide a practical approach for dose adjustments based on uNGAL alongside model-informed precision dosing. Future studies should explore systematic and longitudinal collection of uNGAL and additional biomarkers such as urine TIMP-2*IGFBP-7, urine KIM-1, and serum NGAL and serum cystatin C to further refine PK predictions and should include a rigorous examination of neurotoxicity to ensure cefepime exposure is not excessive. In addition, future studies should prospectively examine the performance of extended vs. standard infusions in achieving PD targets and assess how target attainment predicts clinical outcomes.

## Supplementary Material

1

Supplementary material associated with this article can be found, in the online version, at doi:10.1016/j.ijantimicag.2026.107741.

## Figures and Tables

**Table 1:** Demographics and hospitalisation characteristics of cohort (*n* = 50).

	Demographics				
Age, y, median (IQR)	11.5 (5–17)				
Weight on Study Day 1, kg, median (IQR)	29.4 (20–50)				
Height, cm, median (IQR)	127 (101–151)				
Assigned female sex at birth, *n* (%)	25 (50)				
	Kidney function markers				
SCr-eGFR on PICU admission, mL/min/1.73 m^2^ , median (IQR)	62.2 (41–122)				
uNGAL on PICU admission, ng/mL, median (IQR)	154.5 (118–2065)				
	All	uNGAL < 150	uNGAL ≥ 150	uNGAL < 500	uNGAL ≥ 500
KDIGO SCr-AKI (any Stage) on PICU admission, *n* (%)	34 (68)	13 (26)	21 (42)	20 (40)	14 (28)
KDIGO SCr-AKI (Stage 2 or 3) on PICU admission, *n* (%)	33 (66)	12 (24)	21 (42)	19 (38)	14 (28)
KDIGO SCr-AKI (any Stage) at any point during PICU admission, *n* (%)	36 (72)	15 (30)	21 (42)	22 (44)	14 (28)
KDIGO SCr-AKI (Stage 2 or 3) at any point during PICU admission, *n* (%)	35 (70)	14 (28)	21 (42)	19 (38)	14 (28)
	Illness severity markers				
Mechanically ventilated at any point during study period, *n* (%)	37 (74)				
Received vasopressors at any point during study period, *n* (%)	33 (66)				
Number of organ failures, median (IQR)	2 (2–3)				

The above descriptive statistics include all 50 patients, though one patient was ultimately excluded from PK analyses (see text). Median and standard deviation are reported as variables were not normally distributed. Organ failures considered included cardiovascular, respiratory, neurologic, hematologic, kidney, gastrointestinal, and hepatic failure. Cardiovascular failure was defined as mean arterial pressure (MAP) of <40 or heart rate (HR) of <50 for infants <12 months or a MAP <50 or an HR <40 for patients 12 months or greater, cardiac arrest, or requiring vasoactive infusions for hemodynamic support. Respiratory failure was defined as a respiratory rate (RR) >90 for infants < 12 months, RR >70 for patients 12 months or greater, PaO_2_ <40 mmHg in the absence of cyanotic heart disease, PaCO_2_ >65 mmHg, PaO_2_/FiO_2_ < 250, new mechanical ventilation ( > 24 h post-op), or tracheal intubation for airway obstruction or acute respiratory failure. Neurologic failure was defined as the presence of fixed, dilated pupils or persistent ( >20 min) intracranial pressure >20 mmHg or requiring therapeutic intervention. Hematologic failure was defined as haemoglobin <5 g/dL, WBC <3000 cells/mm^3^, platelets < 20,000/mm^3^, or the presence of disseminated intravascular coagulation (DIC), defined as a prothrombin time >20 s or activated thromboplastin time > 60 s in the presence of a positive fibrinogen split product assay or clinical diagnosis of DIC. Renal failure was defined as a blood urea nitrogen >100 mg/dL, serum creatinine > 2.0 mg/dL, anuria for 24 h, or receipt of any form of dialysis. Gastrointestinal failure was defined as the need for blood transfusions of more than 20 mL/kg due to gastrointestinal haemorrhage. Hepatic failure was defined by the presence of grade 2 or greater hepatic encephalopathy or by a total bilirubin >5 mg/dL and aspartate aminotransferase or lactate dehydrogenase more than twice the upper limit of normal without evidence of haemolysis.

**Table 2:** Population pharmacokinetic parameters of the final cefepime model and bootstrap results.

Parameter	Stochastic approximation		Bootstrap estimates ( *n* = 1000)		
	Estimate	RSE (%)	Median	2.5th percentile	97.5th percentile
*Fixed effects*					
CL = CL_pop_ x (WT/70)^075^ x (eGFR/147.6)^β^ x e^βNGAL^ ^x^ ^NGAL^ ( ^1 or 0^ x *e^ηCl^*					
CL_pop_ (L/h/70kg^0.75^ )	6.38*				
*β*_eGFR_	0.40	25.5	0.37	0.22	0.54
*β*_NGAL_	−0.42	36.9	−0.47	−0.76	−0.13
V1 = V1_pop_ x (WT/70) x^*β*x Cum%FB^ x *^eηVl^*					
V1_pop_ (L/70 kg)	15*				
*β*_Cum%FB_	0.026^*^				
*Q* = Q_pop_ x (WT/70)^0.75^					
Q_pop_ (L/h/70kg^0.75^)	3.65^^*^^				
V2 = V2_pop_ x (WT/70)					
V2_pop_ (L/70 kg)	8.91^*^				
IOV CL	30.3%	16.0	27%	13%	43%
IOV V1	14.2%	73.3	21%	7.6%	130%
*Random effects*					
IIV CL	38.1%^*^				
IIV V1	14.9%^^*^^				
*Error model parameter, proportional only*					
B	31.9%^^*^^				

* Fixed to the value from the cefepime model previously developed and validated by Morales Junior *et al.* [34]. *ηCl* and *ηV1* represent the random-effect parameters for inter-individual variabilities (IIV). The IIV and IOV are expressed as the coefficient of variation (%), calculated as $\sqrt{e^{\omega^{2}}-1}\;x\;100$, where $\omega^{2}$ represents the variance of the random effects.CL, clearance; Cum%FB. cumulative percentage of fluid balance; eGFR, estimated glomerular filtration rate; IIV, inter-individual variability; IOV, inter-occasion variability; Q, intercompartmental clearance; RSE, relative standard error; V1, central volume of distribution; V2, peripheral volume of distribution; WT, body weight in kg.

**Table 3:** Probability of target attainment (PTA) for selected dosing regimens among patients 5 to 40 kg (weight-based dosing), stratified by eGFR.

		uNGAL ≥500 ng/mL			uNGAL <500 ng/mL		
eGFR, mL/ min/1.73 m^2^	PTA for...	100% *fT*>2 mg/L	100% *fT*>8 mg/L	100% *fT*>32 mg/L	100% *fT*>2 mg/L	100% *fT*>8 mg/L	100% *fT*>32 mg/L
**30-60**	50 mg/kg ql2h SI	98.8	87.8	36.8	90.3	56.8	8.7
	50 mg/kg q12h EI	99.5	92.4	44.4	94.6	66.7	12.2
	50 mg/kg q8h SI	100	98.3	73.3	99	87.7	35.1
	50 mg/kg q8h EI	100	99.5	82.7	99.8	94	46.7
	100 mg/kg/d CI	100	100	95.7	100	100	76.5
	150 mg/kg/d CI	100	100	99.7	100	100	95.4
**60-90**	50 mg/kg ql2h SI	96.4	75.2	19.2	79.5	36.7	2.8
	50 mg/kg q12h EI	98.3	82.4	25.4	87.1	49.6	4.5
	50 mg/kg q8h SI	99.9	95.2	54.7	97	74.8	18
	50 mg/kg q8h EI	100	98.1	66.8	99.2	85.5	27.4
	100 mg/kg/d CI	100	100	89	100	100	58.9
	150 mg/kg/d CI	100	100	98.6	100	100	88.3
**90-150**	50 mg/kg ql2h SI	91.3	59.3	9.5	65	21.4	0.8
	50 mg/kg q12h EI	95.3	69.1	13.5	75.5	30.2	1.5
	50 mg/kg q8h SI	99.3	88.5	36.9	92.5	58.6	8.6
	50 mg/kg q8h EI	99.9	94.6	49.1	97.3	73	14.6
	100 mg/kg/d CI	100	100	78.1	100	100	40.6
	150 mg/kg/d CI	100	100	96	100	100	77.1

CI, continuous infusion (over 24 h); eGFR, estimated glomerular filtration rate; EI, extended infusion (over 3 h); PTA, probability of target attainment (% of 10 000 patients achieving PD target); q8h, every 8 h; q12h, every 12 h; SI, standard infusion (over 30 min); uNGAL, urine neutrophil gelatinase-associated lipocalin. Probabilities greater than or equal to 90% are considered to be acceptable for the specific pharmacokinetic/pharmacodynamic target. Patients with uNGAL <500 ng/mL had lower PTA than those with uNGAL >500 ng/mL. More frequent dosing or extended infusions increased PTA. Higher eGFR was associated with lower PTA.

MIC of 2 mg/L represents 100% *f*T > 1x MIC for *Enterobacterales*.

MIC of 8 mg/L represents 100% *f*T > 4x MIC for *Enterobacterales* and 100% *f*T > 1xMIC for *Pseudomonas*.

MIC of 32 mg/L represents 100% *f*T > 4xMIC for *Pseudomonas*.

**Table 4:** Probability of target attainment for selected dosing regimens among patients 40 to 100 kg (fixed dosing), stratified by eGFR.

		uNGAL≥500 ng/mL			uNGAL <500 ng/mL		
eGFR, mL/ min/1.73 m^2^	PT Afor…	100% *f*T>2 mg/L	100% *f*T>8 mg/L	100% *f*T>32 mg/L	100% *f*T>2 mg/L	100% *f*T>8 mg/L	100% *f*T>32 mg/L
**30-60**	2000 mg q12h SI	100	99.1	65.4	99.7	90.9	90.9
	2000 mg q12h EI	100	99.7	74.7	100	95.4	36.6
	2000 mg q8h SI	100	99.2	72.4	99.8	91.5	32.7
	2000 mg q8h EI	100	99.7	80.3	100	95.7	43.1
	4000 mg/day CI	100	100	87.2	100	100	56.8
	6000 mg/day CI	100	100	98.2	100	100	86.7
**60-90**	2000 mg q12h SI	100	97	45.2	99.1	79.3	12.6
	2000 mg q12h EI	100	98.9	56.2	99.7	88.2	19.6
	2000 mg q8h SI	100	97.1	52.7	99.1	80.2	16.4
	2000 mg q8h EI	100	99	63.1	99.7	88.8	24.2
	4000 mg/day CI	100	100	74.4	100	99.9	38.1
	6000 mg/day CI	100	100	94.6	100	100	73.5
**90-150**	2000 mg q12h SI	99.9	91.7	28.4	96.6	63.3	5.4
	2000 mg q12h EI	100	95.9	38.7	98.9	75.8	9.6
	2000 mg q8h SI	99.9	92.3	35	96.7	64.9	7.6
	2000 mg q8h EI	100	96.2	45	98.9	76.7	12.3
	4000 mg/day CI	100	100	58.7	100	99.4	23.4
	6000 mg/day CI	100	100	87.9	100	100	57.5

CI, continuous infusion (over 24 h); eGFR, estimated glomerular filtration rate; EI, extended infusion (over 3 h); PTA, probability of target attainment (% of 10 000 patients achieving PD target); q8h, every 8 h; q12h, every 12 h; SI, standard infusion (over 30 min); uNGAL, urine neutrophil gelatinase-associated lipocalin. Probabilities greater than or equal to 90% are considered to be acceptable for the specific pharmacokinetic/pharmacodynamic target. Patients with uNGAL <500 ng/mL had lower PTA than those with uNGAL >500 ng/mL. More frequent dosing or extended infusions increased PTA. Higher eGFR was associated with lower PTA.

MIC of 2 mg/L represents 100% *f*T > 1x MIC for *Enterobacterales.*

MIC of 8 mg/L represents 100% *f*T > 4x MIC for *Enterobacertales* and 100% *f*T > 1xMIC for *Pseudomonas.*

MIC of 32 mg/L represents 100% *f*T > 4xMIC for *Pseudomonas*.

## Abbreviations:

% *f*T > MIC: percent of time free concentrations exceed minimum inhibitory concentration
AKI: acute kidney injury
AKI stage 1S: subclinical acute kidney injury
CCHMC: Cincinnati Children’s Hospital Medical Center
CL: clearance
CumFB: cumulative percentage of fluid balance
eGFR: estimated glomerular filtration rate
HPLC: high-performance liquid chromatography
ICU: intensive care unit
IOV: inter-occasion variability
IRB: institutional review board
KDIGO: Kidney Disease Improving Global Outcomes
KIM-1: kidney injury molecule-1
MIC: minimum inhibitory concentration
OFV: objective function value
pcVPC: population-corrected visual predictive check
PD: pharmacodynamics
PICU: pediatric intensive care unit
PK: pharmacokinetic
popPK: population pharmacokinetic
PTA: probability of target attainment
Q: intercompartmental clearance
RAI: renal angina index
sAKI: severe acute kidney injury
SCr: serum creatinine
V1: central volume of distribution
V2: peripheral volume of distribution
uNGAL: urine neutrophil gelatinase associated lipocalin
