# Supplementary material for: Urine NGAL adds to serum creatinine in predicting cefepime clearance in critically ill children at high risk of acute kidney injury
Source: Int J Antimicrob Agents. Author manuscript; Available in PMC 2026 Jul 2. (PMC13325201; doi:10.1016/j.ijantimicag.2026.107741)

**Supplemental Files**

**Table S1** – Patients with positive microbiology data.

| Culture Type | Patient Number | Organism(s) |
| --- | --- | --- |
| Blood | 2 | *Micrococcus* |
|  | 22 | *E. Coli*, *Enterococcus hirae, Candida glabrata* |
|  | 42 | *Staphylococcus aureus* |
|  | 51 | *Staphylococcus aureus* |
| Urine | 4 | *Serratia marcescens* |
|  | 43 | *Pseudomonas aeruginosa* |
|  | 47 | *Citrobacter freundii*, *Morganella morganii* |
| Respiratory | 32 | *Pseudomonas aeruginosa* |
|  | 44 | *Candida albicans* |
|  | 48 | *Streptococcus pyogenes* |

**Table S2** – Summary of lowest tested cefepime dose and frequency that reached 90% PTA for selected PD targets. Extended infusions were favored over increasing frequency of dosing.

**5 – 40 kg**

|  | **uNGAL ≥500 ng/mL** | | | **uNGAL <500 ng/mL** | | |
| --- | --- | --- | --- | --- | --- | --- |
| **eGFR, mL/ min/1.73 m^2^** | 100% *f*T >2 mg/L | 100% *f*T >8 mg/L | 100% *f*T >32 mg/L | 100% *f*T >2 mg/L | 100% *f*T >8 mg/L | 100% *f*T >32 mg/L |
| **30-60** | 50 mg/kg q12h, SI | 50 mg/kg q12h, EI | 100 mg/kg/ day, CI | 50 mg/kg q12h, SI | 50 mg/kg q8h, EI | 150 mg/kg/ day, CI |
| **60-90** | 50 mg/kg q12h, SI | 50 mg/kg q8h, SI | 150 mg/kg/ day, CI | 50 mg/kg q8h, SI | 100 mg/kg/ day, CI | No regimen tested |
| **90-150** | 50 mg/kg q12h, SI | 50 mg/kg q8h, EI | 150 mg/kg/ day, CI | 50 mg/kg q8h, SI | 100 mg/kg day, CI | No regimen tested |

**40 – 100 kg**

|  | **uNGAL ≥500 ng/mL** | | | **uNGAL <500 ng/mL** | | |
| --- | --- | --- | --- | --- | --- | --- |
| **eGFR, mL/ min/1.73 m^2^** | 100% *f*T >2 mg/L | 100% *f*T >8 mg/L | 100% *f*T >32 mg/L | 100% *f*T >2 mg/L | 100% *f*T >8 mg/L | 100% *f*T >32 mg/L |
| **30-60** | 2000 mg q12h, SI | 2000 mg q12h, SI | 6000 mg/day, CI | 2000 mg q12h, SI | 2000 mg q12h, SI | No regimen tested |
| **60-90** | 2000 mg q12h, SI | 2000 mg q12h, SI | 6000 mg/day, CI | 2000 mg q12h, SI | 4000 mg/day, CI | No regimen tested |
| **90-150** | 2000 mg q12h, SI | 2000 mg q12h, SI | No regimen tested | 2000 mg q12h, SI | 4000 mg/day, CI | No regimen tested |

CI, continuous infusion (over 24 hours). eGFR, estimated glomerular filtration rate. EI, extended infusion (over 3 hours). PTA, probability of target attainment (% of 10,000 patients achieving PD target). q8h, every 8 hours. q12h, every 12 hours. SI, standard infusion (over 30 minutes). uNGAL, urine neutrophil gelatinase-associated lipocalin.

MIC of 2 mg/L represents 100% *f*T > 1x MIC for *Enterobacertales*

MIC of 8 mg/L represents 100% *f*T > 4x MIC for *Enterobacterales* and 100% *f*T > 1xMIC for *Pseudomonas*

MIC of 32 mg/L represents 100% *f*T > 4xMIC for *Pseudomonas*

**Figure S1**. Population (left) and individual (right) model predictions versus observations for cefepime concentrations (mg/L). The solid line represents the line of unity (y=x).


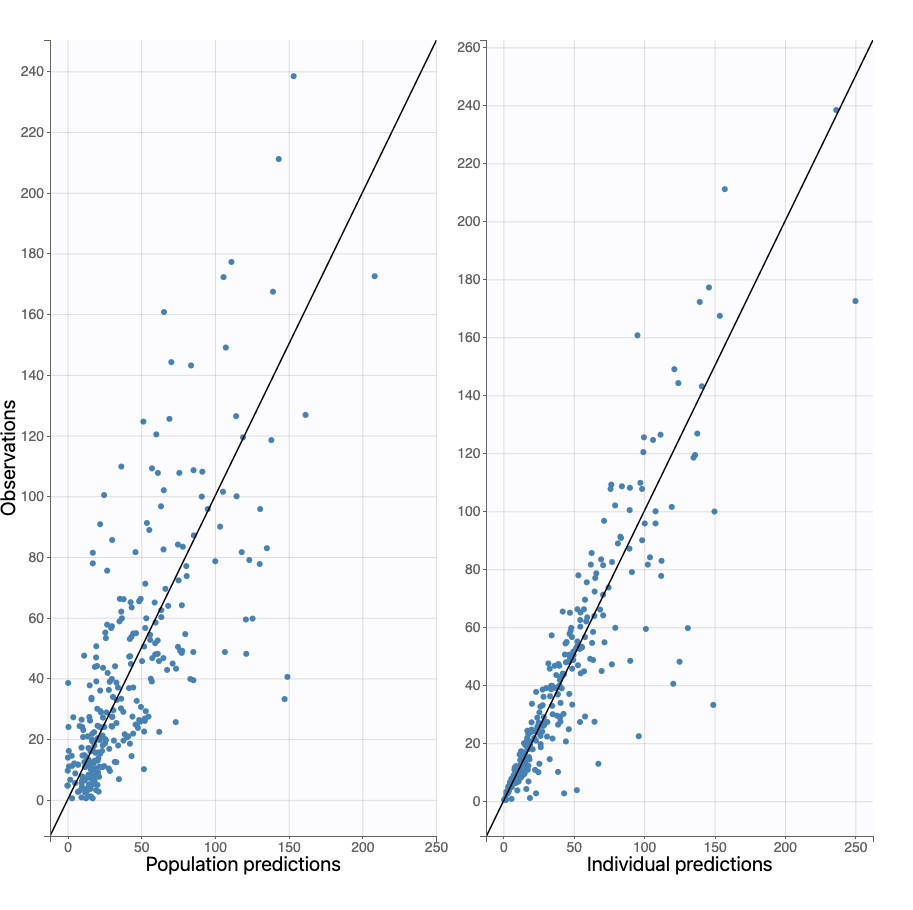


**Figure S2**. Residual plots of the final cefepime model. Left panel: individual weighted residuals (IWRES) versus time (time zero = first patient sample) in hours. Right panel: IWRES versus individual-predicted concentrations. Concentrations are in mg/L.


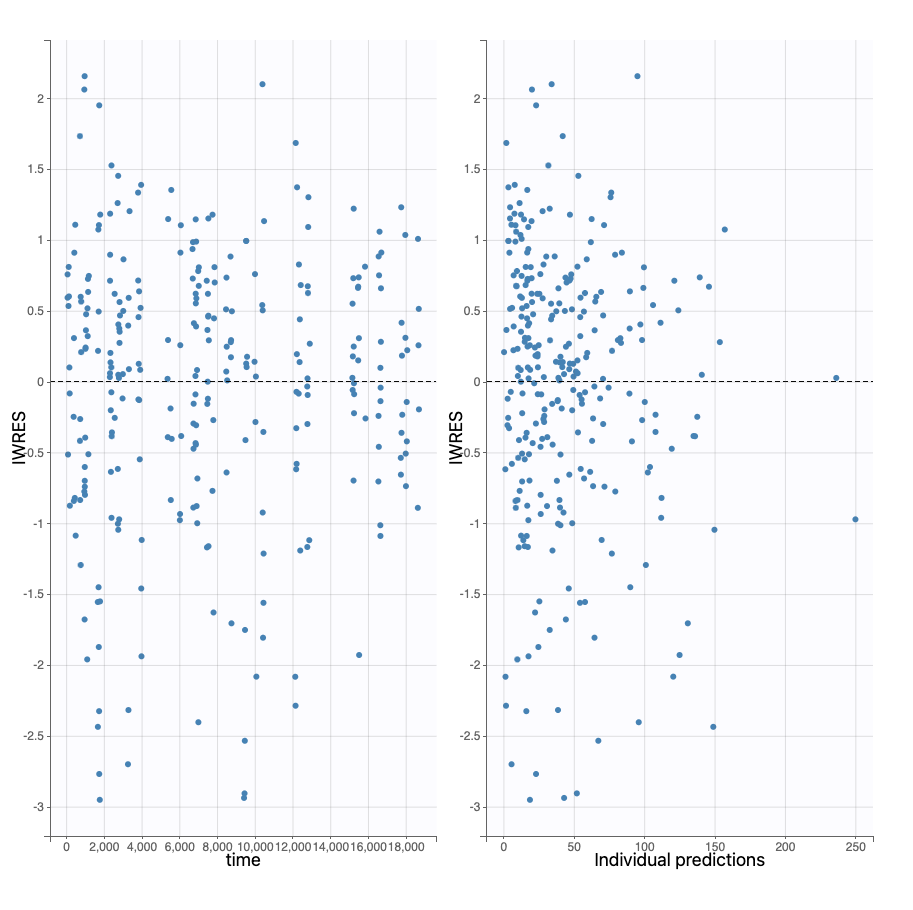


**Figure S3**. Prediction-corrected visual predictive check (pcVPC) for cefepime concentrations. Blue dots represent observed concentrations. Solid lines represent the median, 5th and 95th percentile of the observed data, while shaded areas show the 95% prediction intervals (n=1,000) generated from the model.


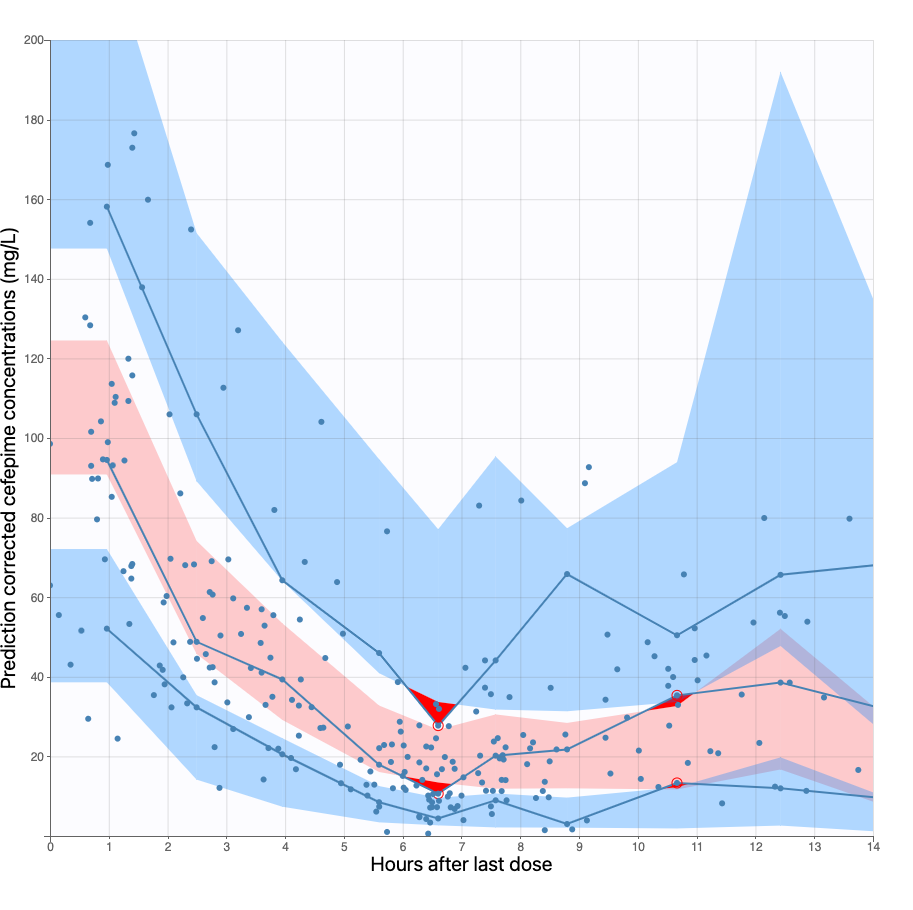

Supplement: 1 [file NIHMS2177825-supplement-1.docx]
